# Different Morphology of Branching Neovascular Network in Polypoidal Choroidal Vasculopathy: A Swept-Source Optical Coherence Tomography Angiography Study

**DOI:** 10.3390/jcm12030742

**Published:** 2023-01-17

**Authors:** Lulu Chen, Mingzhen Yuan, Lu Sun, Youxin Chen

**Affiliations:** 1 Department of Ophthalmology, Peking Union Medical College Hospital, Chinese Academy of Medical Sciences, Beijing 100730, China; 2 Key Laboratory of Ocular Fundus Diseases, Chinese Academy of Medical Sciences, Beijing 100730, China; 3 Beijing Ophthalmology and Visual Sciences Key Laboratory, Beijing Tongren Eye Center, Beijing Tongren Hospital, Capital Medical University, Beijing 100730, China

**Keywords:** polypoidal choroidal vasculopathy, branching neovascular network, polypoidal lesion, classification, Bruch’s membrane

## Abstract

**Purpose:** To evaluate the classification system of branching neovascular network (BNN) morphology in polypoidal choroidal vasculopathy (PCV) patients based on swept-source optical coherence tomography (SS-OCT) and swept-source optical coherence tomography angiography (SS-OCTA), and analyze the morphological features in each group as potential prognostic features. **Methods:** A total of 32 PCV eyes were included in this retrospective study. SS-OCT and SS-OCTA images of 6 mm × 6 mm centered on the foveal of each eye were analyzed. PCV cases were classified into three types (“trunk”, “glomeruli”, and “stick” type) based on the morphological features of BNN. OCT and OCTA features were compared among the three groups. The correlation of OCT/OCTA features with visual acuity at 12 months after anti-VEGF treatment was also analyzed. **Results:** Type 1 group had the largest BNN area and the largest numbers of polypoidal lesions. Type 2 group has the largest pigment epithelial detachment (PED) area, PED volume, subretinal fluid (SRF) area, and SRF volume. Type 3 group had better baseline BCVA, the smallest BNN area, the smallest PED size, and the smallest SRF size. Type 1 was also featured by a clear break on Bruch’s membrane which corresponded to the origin of neovascular tissue. BCVA at 12 months was not significantly different among groups. Baseline BCVA and baseline central macular thickness were correlated with the final BCVA. **Conclusions:** The current classification system based on BNN morphology on SS-OCTA was highly applicable and revealed distinct characteristics in each group. The BNN type was not correlated with BCVA at 12 months after treatment.

## 1. Background

Polypoidal choroidal vasculopathy was a clinical entity first described by Yannuzzi in 1982 [1] and has been considered a distinct entity from neovascular age-related macular degeneration (AMD) [2,3]. The prevalence of PCV in different populations of AMD patients ranges from 8% to more than 50%, with a higher preponderance in Asian patients. The prevalence of PCV in the entire population ranges from 0.04% in the European to approximately 0.5% in the Chinese population [4,5,6]. Though not fully understood, growing clinical, imaging, and animal studies have provided insights into the pathogenesis of PCV. Many studies have proven the important role of vascular endothelial growth factor (VEGF) in the pathogenesis of AMD. However, the concentration of VEGF in aqueous humor was lower in PCV compared with typical AMD. Genetic studies found single-nucleotide polymorphisms (SNP) including CFH, ARMS2, and HTRA1 were associated with PCV phenotype [7]. Recent OCT studies demonstrated choroidal thickening and other structural changes in the choroidal layer in PCV, and many studies have attributed PCV to pachychoroid spectrum diseases [8,9]. With the accumulation of knowledge about PCV, it appears that PCV has unique characteristics and has grasped the interest of researchers. PCV is known to be characterized by polypoidal lesions and branching vascular networks (BVN) on indocyanine green angiography (ICGA) [10,11]. However, ICGA is an invasive and time-consuming test, which limits its use in the clinic. With the development of imaging modalities, recent studies have come up with new diagnostic criteria with non-invasive multi-modal imaging systems and evaluated the feasibility of these criteria in PCV diagnosis [12,13,14,15].

Among all the approaches, optical coherence tomography (OCT) provided high-resolution images with specific imaging features closely related to PCV. It has become the mainstay imaging method in the diagnosis and follow-up of PCV. Recently, the Asia–Pacific Ocular Imaging Society (APOIS) PCV Workgroup proposed a non-ICGA diagnostic criteria using spectral-domain OCT(SD-OCT) which achieved high accuracy in PCV diagnosis. The workgroup has also recommended the terms polypoidal lesion and branching neovascular network (BNN) to replace the terms polyps and BVN. The consensus has brought new insight into the utility of OCT in the diagnosis and follow-up of PCV [16]. Compared with OCT, OCTA revealed more information about the neovascular structures. The current devices of swept-source OCT and OCTA imaging at 1050 nm have the advantage of having better penetration into the choroidal region, allowing the study of abnormalities including CNVs. Additionally, imaging at this wavelength provides the least water (vitreous)-induced dispersion to OCT signal, thereby enhancing the image quality. These advantages of the SS-OCT system make it a promising tool for diagnostic retinal imaging, not only for humans but also for vertebrates in veterinary ophthalmic research [17,18,19]. Recent research on PCV has shown that current SS-OCT and OCTA devices facilitated the detection of BNNs and polypoidal lesions with details better than SD-OCT [20,21].

Efforts have been made to understand the pathogenesis of PCV, and several classification systems have been raised to characterize PCV into different subtypes. Kawamura categorized PCV into two types based on the presence of feeder vessels and draining vessels on ICGA [22]. Jang divided PCV into the T-PCV group and P-CNV group according to the presence of features of pachychoroid or AMD [23]. Baek’s study focused on the morphology of choroidal vessels [24]. Liu and Venkatesh classified PCV cases into subtypes based on the thickness of the choroid layer [25,26]. Those studies reported different features and treatment outcomes in different types of PCV, but a consensus has not been achieved. As an important component of PCV, BNN has raised interest among researchers. A recent study by Huang et al. proposed a new classification system based on the morphology of BNN on OCTA and demonstrated that BNN morphology has the potential to prognosticate outcomes in PCV patients [27,28]. However, the treatment strategy in previous studies included anti-VEGF and photodynamic therapy, which was not applicable in the current situation. In this study, the authors aimed to analyze the morphological features of each type of PCV with OCTA using Huang’s classification system. In addition, the authors aimed to evaluate the visual outcome at 12 months with anti-VEGF treatment in correlation to different BNN morphology and biomarkers on OCT/OCTA.

## 2. Methods

This retrospective study included patients who were diagnosed with PCV by ICGA and were followed for 12 months from January 2019 to October 2021 at the Department of Ophthalmology at Peking Union Medical College Hospital, Beijing, China. The study followed the tenets of the Declaration of Helsinki and was approved by the Institutional Board of Peking Union Medical College Hospital. Informed consent was obtained from all participants. All the patients enrolled were evaluated with comprehensive ophthalmic examinations, including best corrective visual acuity (BCVA), slit-lamp examination of the anterior segment and fundus, and swept-source OCT/OCTA (VG200; SVision Imaging, Ltd., Luoyang, China) at each visit. Fluorescein angiography (FA) and ICGA (Spectralis HRA, Heidelberg Engineering, Germany) images were captured at baseline. The inclusion criteria were as follows: (1) eyes with the clinical diagnosis of PCV by two independent retinal specialists based on the ICGA criteria used in the EVEREST study [29], and (2) treatment-naïve PCV eyes. The exclusion criteria were high myopia (≥6.00 diopters), previous vitrectomy surgery, diabetic retinopathy, presence of extensive subretinal hemorrhage that prevented adequate OCTA evaluation, and other concomitant retinal diseases. The patients received intravitreal injections of anti-vascular endothelial growth factor (VEGF) agent (Ranibizumab, Conbercept, or Aflibercept) with a 3 + PRN protocol in the study period. No eye received photodynamic treatment (PDT) during the observation period.

ICGA images were evaluated by two different retinal specialists to identify polypoidal lesions in the early phase and the presence of BNNs. The SS-OCT/OCTA instrument used a central wavelength of 1050 nm (990–1100 nm full width) and an A-scan rate of 200,000 per second. A 6 mm × 6 mm OCTA image centered on the lesion was acquired for each enrolled eye. OCTA volumes of the retina and choroid were automatically segmented by the built-in software and segment errors were corrected manually by one retinal specialist for further evaluation. 

The central macular thickness (CMT) and central choroidal thickness (CCT) were calculated in the inner 1 mm circle of the Early Treatment of Diabetic Retinopathy Study (ETDRS) chart. The maximum height of PED was measured manually as the maximum distance between the Bruch’s membrane and pigment epithelial layer for each eye. The VG200D’s van Gogh software uses artificial intelligence algorithms based on deep learning to identify each layer of retina. In cases of segment errors, manual correction can be applied. After a precise delineation of the RPE and Bruch’s membrane, the contours of pigment epithelial detachment (PED) were recognized automatically in each B-scan. Area calculation in 2D and volume calculation in 3D were then performed automatically and simultaneously. Features of PEDs were recorded at the same time.

**Figure 1:**
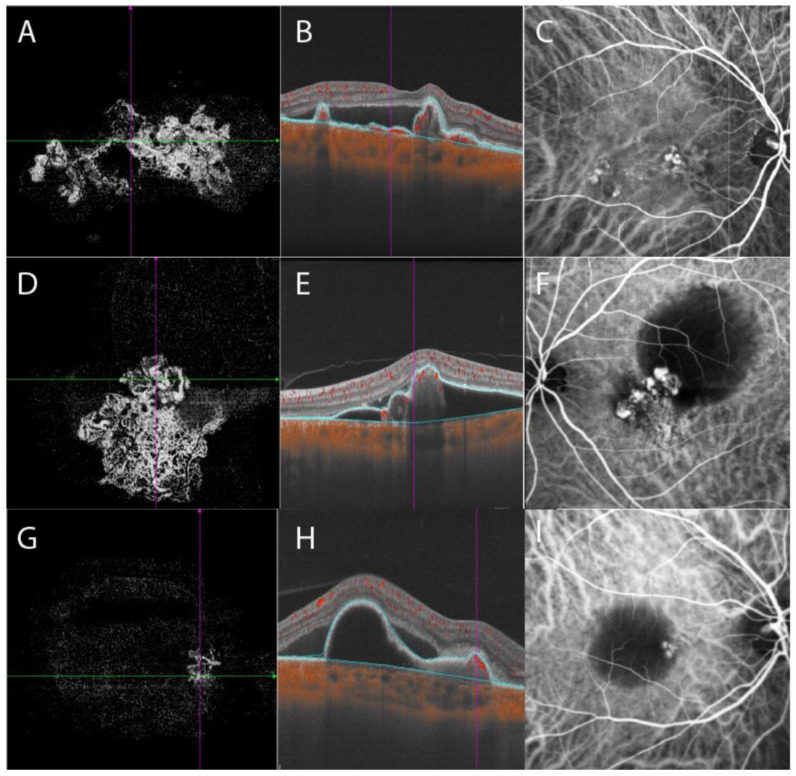
Three BNN subtypes were demonstrated with OCTA and ICGA. BNN morphologies within the PED layer were demonstrated in the left column. B-scan OCTA images with blue lines delineating the PED layer were shown in the middle column. ICGA images were shown in the right column. En face OCTA of type 1 BNN, characterized by the presence of a major “trunk” or “stem”. The neovascular vessels radiated and spread toward the periphery area with tangled vessels forming polypoidal lesions at the edge of BNN (**A**). B-scan OCTA showed peaked PED with internal flow signals and subretinal fluid (**B**). ICGA showed multiple polypoidal lesions with a BNN (**C**). Type 2 BNN was featured by a cluster of interconnected neovascular vessels without a stem or trunk. Polypoidal lesions were embedded inside the BNN or at the edge of the branches of neovascular vessels (**D**). B-scan OCTA showed PED with notches and internal flow signals (**E**). A cluster of polypoidal lesions connected with BNN was shown on ICGA, accompanied by a hypofluorescent area representing PED (**F**). Type 3 BNN had relatively smaller BNN on en face OCTA (**G**). B-scan OCTA showed notched PED with internal flow signals and subretinal fluid (**H**). Polypoidal lesions with small BNN were shown on ICGA (**I**). BNN, branching neovascular network; OCTA, optical coherence tomography angiography; ICGA, indocyanine green angiography; PED, pigment epithelial detachment.

Both the en face and cross-sectional B-scans of OCTA were reviewed to identify all polypoidal lesions and BNNs. Polypoidal lesions were recognized as foci with flow signals that were associated with dome-shaped, peaked PEDs on B-scans. BNNs were identified as vascular networks on en face slabs associated with double layer sign on B-scans. The segmentation of PED layer was reviewed for better visualization of polyps and BNN detection. PED layer was defined as the volume from the border of retinal pigment epithelial to the border of Bruch’s membrane. The PED layer was segmented automatically by the installed software and segment errors were corrected manually (Figure 1). The total lesion size of the PCV on en face OCTA was measured using the “area tool” installed in the instrument. Subretinal fluid area and volume were measured automatically with the installed software. The software described above identify each layer of retina. After a precise delineation of the margin of neurosensory retinal and RPE, the area of subretinal fluid was recognized precisely in each B-scan. Area calculation in 2D and volume calculation in 3D were then performed automatically and simultaneously. Choroidal vascularity index (CVI) refers to the ratio of the volume of the large and medium choroidal vessels to the volume of the choroid. The software uses artificial intelligence algorithms to identify the contours of the large and medium choroidal vessels in the B-scans and then forms the morphology of vessels through three-dimensional reconstruction to quantify large and medium-sized choroidal blood vessels. CVI value within the inner 1 mm circle of the ETDRS chart was recorded. The SS-OCTA images were overlaid on the ICGA images to determine the location of polypoidal lesions. 

**Figure 2:**
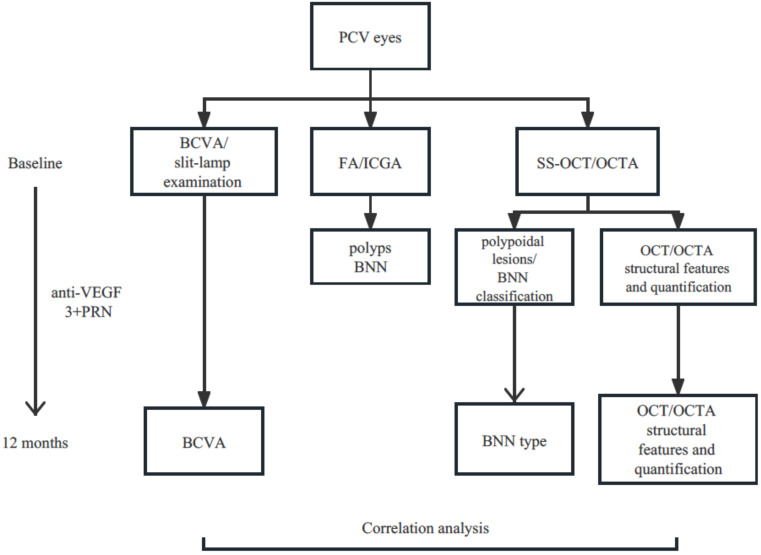
Flow chart demonstrating a simplified study protocol. PCV, polypoidal choroidal vasculopathy; BCVA, best corrected visual acuity; FA, fluorescein angiography; ICGA, indocyanine green angiography; SS-OCT, swept source optical coherence tomography; OCTA, optical coherence tomography angiography; BNN, branching neovascular network; VEGF, vascular endothelial growth factor; PRN, pro re nata.

The morphology of BNN images under manual segmentation was reviewed and cases with similar features were gathered together into three different types of BNNs, as Huang’s group, has proposed [18] by two retinal specialists. Type 1, the “Trunk” type: one or more trunks sprouting from the same origin which then radiated to form more branches towards the periphery. Type 2, the “Glomeruli” type: a group of vascular structures interconnecting with each other to form a glomeruli-like structure without any clear main stem. Type 3, the “Stick” type: a few thin and fine vascular structures without complicated interconnections or clear stem (Figure 1). The Cohen’s Kappa between graders was then evaluated. A third retinal specialist confirmed the disparity in the classification of BNN type. Figure 2 demonstrates a simplified study protocol.

## 3. Statistical Analysis

Statistical analysis was performed using the software SPSS 25.0 (IBM, Chicago, IL, USA). The categorical data were evaluated with the chi-square test or Fisher exact test. Continuous variables were presented as mean and standard deviation. The normality of data was examined by the Shapiro–Wilk test. For data that conform to a normal distribution, analysis of variance (ANOVA) was applied, and LSD or Games–Howell tests were conducted for post hoc tests. Kruskal–Wallis H test was applied for data that were not normally distributed. Post hoc analysis was applied with Bonferroni correction. Paired *t*-test was applied for comparison of BCVA and OCT/OCTA metrics at baseline and 12 months after treatment in each group. Multivariate linear regression was applied to analyze the correlation of OCT/OCTA features with BCVA at 12 months. *p* < 0.05 was considered statistically significant.

## 4. Results

A total of 32 eyes diagnosed with PCV from 31 patients were included in this study. The average age of the patients was 66.0 ± 8.0 years old and 51.6% were males. The baseline BCVA was 0.49 ± 0.35 (Log MAR) and the average number of anti-VEGF injections was 5.0 ± 2.0. Ten eyes were treated with Ablifercept, eleven eyes were treated with Conbercept, seven eyes were treated with Ranibizumab, three eyes were initially treated with Ranibizumab and then switched to Ablifercept, and one eye was initially treated with Conbercept and then switched to Ablifercept. The BNNs were detected in all 32 eyes (100%) on SS-OCTA images, while the BNN detection rate was 90.9% on ICGA. A total of 100 polypoidal lesions were detected on ICGA, which could all be found on SS-OCTA. Another nine polypoidal lesions were detected on SS-OCTA but were not detected on ICGA, and were correlated with peaked or notched PEDs with internal flow signal on OCTA B-scans. 

**Figure 3:**
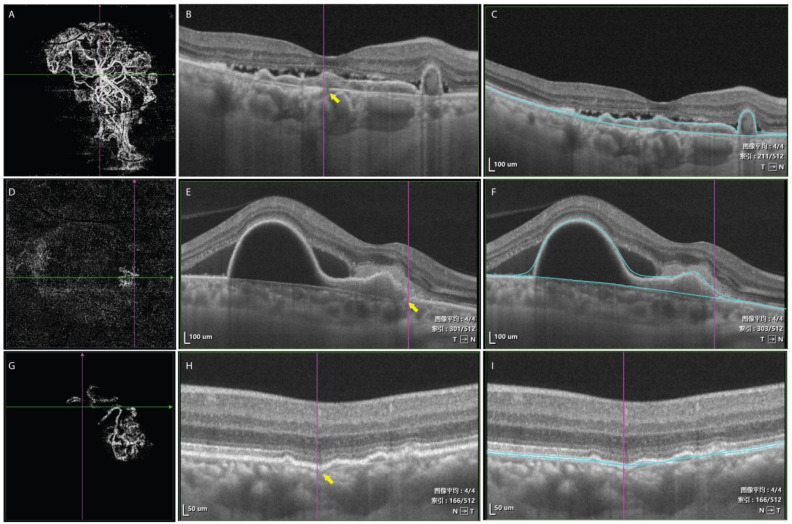
En face scan of OCTA showed a large BNN with a main stem in type 1 BNN (**A**). A clear break was shown on B-scan OCT corresponding to the location of the origin of the BNN on en face OCTA (yellow arrow, (**B**)). Blue lines showing the corresponding segmentation of PED layer (**C**). En face scan of OCTA demonstrating BNN without a main stem (**D**). The line representing Bruch’s membrane on OCT was irregular but without a clear break (yellow arrow, (**E**)). Blue lines showing the corresponding segmentation of PED layer (**F**). En face scan of OCTA demonstrating BNN without a main stem (**G**). The Bruch’s membrane slightly curved toward the choroid layer and became increasingly obscure (**H**). Blue lines showing the corresponding segmentation of PED layer (**I**). 图像平均, average image; 索引, index. T, temporal; N, nasal.

Eleven eyes (34.4%) were classified into type 1 group, six eyes (18.8%) into type 2 group, and 15 eyes (46.9%) into type 3 group. The Cohen’s Kappa between graders in BNN classification was 0.902 (95%CI 1.031–0.733, *p* < 0.001). Baseline evaluation of BCVA, central choroidal thickness, PED maximum height, PED area, subretinal fluid volume, and choroidal vascular index were comparable among groups (Table 1). Central macular thickness in type 2 group was thicker than that in type 3 group (type 2 group 395 ± 140 μm, type 3 group 281 ± 81 μm, *p* = 0.049, post hoc LSD test). PED volume in type 2 group was larger than that in type 1 group and type 3 group, but the difference was not statistically significant (*p* = 0.082). The BNN lesion area was significantly different among groups and type 1 group had the largest lesion area of 6.827 ± 3.115 mm^2^ (type 2 group 3.722 ± 1.997 mm^2^, type 3 group 1.710 ± 1.213 mm^2^, *p* < 0.001). The average number of polypoidal lesions was significantly different among groups (5.09 ± 3.18 in type 1 group, 3.00 ± 1.67 in type 2 group, and 2.33 ± 1.50 in type 3 group, *p* = 0.027), and type 1 group had more polypoidal lesions than the other two types. In type 1 BNN, a clear break of the Bruch’s membrane can be found on B-scan OCT, which corresponds to the origin of the main trunk of neovascular vessels on en face OCTA. In type 2 and 3 BNN, there was no clear break on Bruch’s membrane. The Bruch’s membrane was either intact or slightly curved towards the choroid layer (Figure 3).

**Figure 4:**
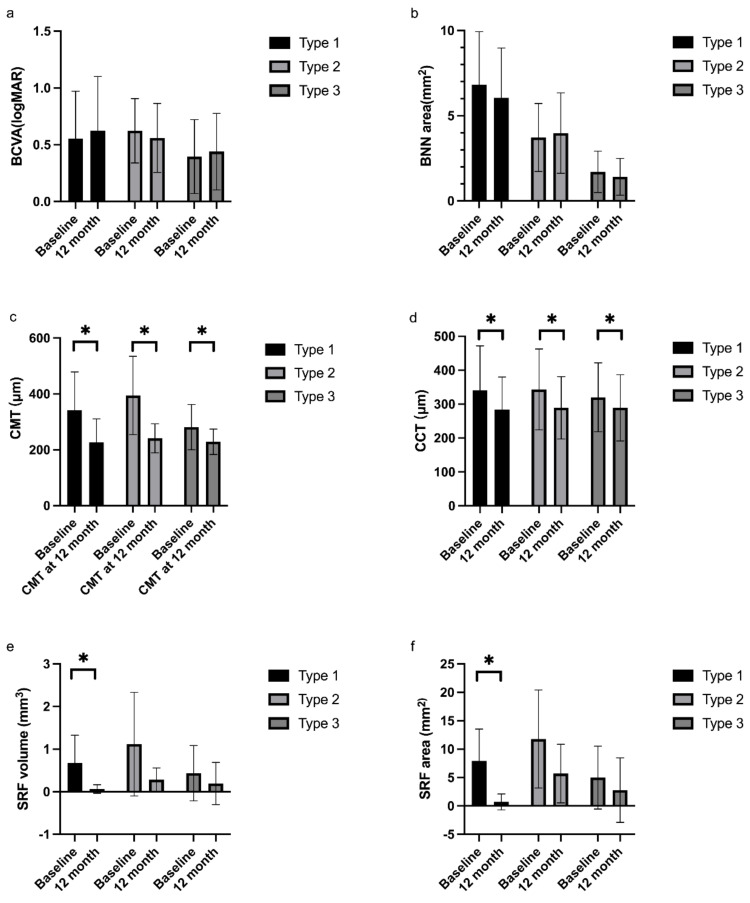
Comparison of BCVA and OCT/OCTA parameters in three BNN subtypes. There was no statistically significant difference in BCVA and BNN area after treatment at 12 months in each group (**a**,**b**). Central macular thickness decreased significantly in all three groups after anti-VEGF treatment at 12 months (**c**). Central choroidal thickness decreased significantly in all three groups after anti-VEGF treatment at 12 months (**d**). SRF volume decreased in all groups at 12 months but was statistically only in type 1 BNN (**e**). SRF area decreased in all groups at 12 months but was statistically only in type 1 BNN (**f**). BCVA, best corrected visual acuity; BNN, branching neovascular network; CMT, central macular thickness; CCT, central choroidal thickness; SRF; subretinal fluid. * Paired *t*-test, *p* < 0.05.

The average number of intravitreal anti-VEGF injections was 5.09 ± 2.26 in type 1 group, 4.67 ± 1.63 in type 2 group, and 4.33 ± 1.76 in type 3 group (*p* = 0.617) in the follow-up period of 12 months. The BCVA at 12 months was not significantly different compared with baseline in each group (0.63 ± 0.48 in type 1 group, and 0.56 ± 0.30 in type 2 group, 0.44 ± 0.34 in type 3 group, all *p* > 0.05). Central macular thickness decreased significantly in all groups compared with baseline (type 1 group 341 ± 138 μm vs. 226 ± 84 μm *p* = 0.034; type 2 group 395 ± 140 μm vs. 241 ± 52 μm *p* = 0.012; type 3 group 281 ± 81 μm vs. 229 ± 45 μm *p* = 0.022). Central choroidal thickness decreased significantly in all groups compared with baseline (type 1 group 341 ± 131 μm vs. 284 ± 96 μm *p* = 0.039; type 2 group 344 ± 119 μm vs. 289 ± 92 μm *p* = 0.027; type 3 group 320 ± 102 μm vs. 289 ± 98 μm *p* = 0.048). Subretinal fluid area and volume decreased in all three groups; however, the differences were statistically significant only in type 1 group (Figure 4). VI, BVN area, PED area, PED volume, and maximum PED height were not significantly different compared with those at baseline (all *p* > 0.05). Comparison of BCVA and OCT/OCTA structure parameters among three groups at 12 months were shown in Table 2. 

The correlation of baseline structural biomarkers and BNN morphology on OCT/OCTA with BCVA at 12 months after treatment was analyzed with multivariate linear regression. Baseline BCVA and baseline central macular thickness were correlated with the final BCVA (Table 3). BNN morphology was not correlated with the BCVA after treatment at 12 months. 

## 5. Discussion

In the present study, the authors classified the PCV based on BNN morphology with a previous classification system and evaluated SS-OCT/OCTA features in each subtype. OCT/OCTA features of each type were well-evaluated and correlated with visual outcome after anti-VEGF treatment at 12 months. 

In our study, BNN can be detected in all cases and more polypoidal lesions were detected on OCTA than on ICGA. Previous studies have shown a varied detection rate of BNN with OCTA from 70% to 100% [27,30,31,32], while the sensitivity for detection of polyps was 40.5% to 100% [32,33,34]. In our study, the high detection rate of BNNs with SS-OCTA was attributable to the high penetration and resolution of the device. Furthermore, we detected nine more polypoidal lesions with OCTA compared with ICGA, and those lesions corresponded to notched or peaked PEDs with internal flow signal. The SS-OCTA could reveal polypoidal lesions clearly when the lesions were located under large PED or in the presence of submacular hemorrhage, which could be the main reason that these lesions were not visible on ICGA. 

A rising interest has focused on PCV classification in recent years and some researchers have proposed different criteria based on distinguished imaging features with a variety of modalities. Coscas and colleagues differentiated PCV into idiopathic PCV and NV-AMD-related polyps [35]. Based on this classification criteria, Dijk and colleagues further categorized Caucasian PCV into PCV in the context of neovascular AMD, PCV with non-polypoidal type 1 CNV/BVN, and idiopathic PCV [36]. These studies revealed differences between Caucasian and Asian PCV patients and presumed that Caucasian PCV patients were more likely to be associated with an AMD background than Asian patients. Jang et al. differentiated PCV into typical PCV(T-PCV) and polypoidal choroidal neovascularization(P-CNV) and described the different subfoveal fibrosis rates and responses to anti-VEGF treatment in the two subtypes [23]. Another study categorized PCV into three subtypes according to the sub-foveal choroidal thickness [26] and found similar treatment outcomes among the three groups. Despite polypoidal lesions, BNN was another distinctive component of lesions in PCV and some other researchers also tried to categorize PCV based on features of BNNs with clinical relevance. Kawamura et al. divided PCV into two groups according to the presence of feeder vessels and draining vessels on ICGA [22]. Tan’s group classified PCV into three types based on the presence of interconnecting channels on ICGA and leakage on FA [37]. This system has been verified in later studies and has shown potential in predicting visual prognosis [38,39,40]. Huang et al. divided PCV into three forms (trunk, glomeruli, and stick forms) with morphological features of BNN on SS-OCTA and reported a high correlation between their classification system with Tan’s system [27]. However, only a few studies have focused on the clinical features and treatment response in PCV patients with OCTA-based BNN subtypes. 

In our study, we divided PCV cases into three types based on Huang’s system and further explored the SS-OCTA features of each group with a follow-up of 12 months. Two graders classified all enrolled OCTA images and the Cohen’s Kappa was 0.902, indicating that the classification system was highly repeatable. 

According to Huang’s study, type 1 PCV tends to have poorer baseline BCVA and larger total lesion size. Type 1 PCV (trunk type) in our study agreed with those in Huang’s study, and those PCVs had the largest baseline BNN area and the most polypoidal lesions. The main stem of the neovascular vessel originated from a clear break in Bruch’s membrane and radiated into smaller branches pointing toward the periphery area. Polypoidal lesions were tangled at the edge of the branches like fruits on a tree (Figure 1). The central macular thickness was thicker in type 2 at baseline, though the difference was not statistically significant. Type 2 group also had larger PED area, PED volume, subretinal fluid area, and subretinal fluid volume, though not statistically significant. Type 3 had better baseline BCVA, though not statistically significant, and thinner central retinal thickness. PCV cases in this group have the smallest BNN lesions and least polypoidal lesions at baseline. The small lesion size could be the reason for the relatively better vision in this group of patients. In Huang’s research, type 3 PCV had a thicker subfoveal choroidal layer and they tended to associate PCVs in this group with pachychoroid spectrum disease. However, in our study, baseline SFCT in type 3 PCV did not differ from the other two types, but PCV in this group had a smaller PED area and SRF area. 

Another interesting feature in type 1 BNN was the break in Bruch’s membrane on B-scan OCT. In Kawamura’s study, a break in Bruch’s membrane was detected with SD-OCT in type 1 PCV (with feeder vessel and draining vessel on ICGA). The breaking point was correlated to the in-growth site of neovascular vessels. The line representing Bruch’s membrane was straight and thin. In type 2 PCV (without feeder vessel and draining vessel on ICGA), the line representing Bruch’s membrane was intact but irregular. It curved downward and became obscure and disappeared at the site at which the network filling began [22]. However, this study was based on findings from SD-OCT and ICGA, and the morphology of neovascular tissues was not elucidated. In our study, BNNs were well elucidated with SS-OCTA, which enabled us to better reveal the origin of BNNs. Type 1 BNN in our study shared many similarities with type 1 in Kawamura’s study. The break in Bruch’s membrane on B scan was located on en face OCTA image and corresponded to the origin of the main trunk of BNN. In type 2 and 3 BNNs, there were no obvious breaks in Bruch’s membranes and the Bruch’s membranes were either intact or slightly curved towards the choroid layer. The origin of BNNs in type 2 and 3 was not as obvious as in type 1 BNN, and they shared similarities with type 2 PCV in Kawamura’s study.

After 12 months of follow-up, central macular thickness and central choroidal thickness decreased significantly in all groups. SRF volume and SRF area decreased significantly in type 1 PCV, but the visual outcome was not significantly improved. BCVA at 12 months was the worst in type 1 group, but the difference was not statistically significant. In Huang’s study, the recurrence rate of SRF was higher in type 2 PCV and the visual outcome was worst in type 1 PCV. A later study concluded that visual improvement was more significant in type 2 PCV with combined treatment of anti-VEGF and PDT and the morphology of BNN was correlated with BCVA at 12 months after treatment [28]. The authors attributed the poor vision in type 1 PCV to the involvement of fovea with larger lesion size and the leakage of BNN. The treatment strategy was different in our study and the improvement in BCVA was not significant in each group. Multivariate regression analysis revealed that BCVA at 12 months was only correlated with baseline BCVA and baseline central macular thickness. The morphology of BNN was not correlated with the BCVA after treatment with anti-VEGF injections. 

The intactness of the fovea was crucial to the central vision. In type 1 group, the area of BNN was large and there was a higher chance that the fovea was covered by the BNN that spread out. The strong trunk and stem in type 1 group might be hard to shrink in response to anti-VEGF treatment and might even form scar tissues which could leave poor function of the fovea. In type 3 group, the BNN was small and there was a smaller chance that the BNN would invade the fovea. The fine and thin neovascular branches shrink after anti-VEGF treatment and the function of the fovea restores once the fovea becomes dry. The type 2 group was more like a mixture of strong and thin branches. The thin branches are likely to shrink in response to anti-VEGF agents but the strong branches are hard to shrink.

However, our study only enrolled a limited number of PCV cases with a relatively short follow-up period; the morphological changes of BNN and visual outcome in the long term need to be clarified in the future.

Compared with previous classification studies in PCV, our study evaluated the PCV classification system with the advanced SS-OCT/OCTA device, which detects polypoidal lesions and BNNs in detail with high resolution. Besides, the study area was 6 mm × 6 mm in our study, larger than Huang and Ma’s studies. The larger study area helped us to detect more polypoidal lesions and exhibited the lesion on a full scale. Besides, we only enrolled treatment-naïve patients who were then treated only with anti-VEGF drugs. The different study protocols might explain some of the discrepancies in our results to Huang’s and Ma’s. There were several limitations in our study. The major one was the retrospective nature of the study with limited study cases. Another limitation is that all the cases enrolled were based on an Asian population. Caution should be exercised when generalizing results into Caucasians/Whites, as PCV tends to manifest differently between the two ethnicities [41,42]. Besides, some patients may not get enough injections of anti-VEGF drugs due to the pandemic of COVID-19 during the follow-up period. The study area was designed to be 6 mm × 6 mm; patients with larger lesion sizes were not included in the present study. The observation period was only 12 months in the current study; studies with a longer follow-up period would be helpful to reveal the long-term outcome of each type of PCV and the possible morphological changes. 

## 6. Conclusions

To sum up, our study showed the current classification system based on BNN morphology on SS-OCTA was highly applicable and revealed distinct characteristics in each group. Similar treatment outcomes were achieved in each group with different morphological characteristics. Baseline BCVA and baseline central macular thickness, rather than the morphological features revealed by SS-OCTA, could be useful prognostic factors in PCV patients.

## Figures and Tables

**Table 1:** Baseline characteristics among 3 PCV subgroups.

	Type1 (n = 11)	Type2 (n = 6)	Type3 (n = 15)	*p* Value
Age (year)	65.8 ± 6.6	66.0 ± 7.8	65.5 ± 9.3	0.989
Gender (male%)	45%	50%	60%	0.753
BCVA (logMAR)	0.555 ± 0.416	0.624 ± 0.283	0.397 ± 0.325	0.268
OCT-CMT (μm)	341 ± 138	395 ± 140	281 ± 81	0.117 ^$^
OCT-CCT (μm)	341 ± 131	344 ± 119	320 ± 102	0.995
OCTA-BNN area (mm^2^)	6.827 ± 3.115	3.722 ± 1.997	1.710 ± 1.213	<0.001 *
OCTA-polyp number	5.09 ± 3.18	3.00 ± 1.67	2.33 ± 1.50	0.027 **
OCT-PED height (μm)	400 ± 129	394 ± 198	304 ± 191	0.105
OCT-PED volume (mm^3^)	1.088 ± 0.788	1.997 ± 1.714	0.802 ± 1.233	0.082
OCT-PED area (mm^2^)	12.879 ± 5.527	15.872 ± 10.097	8.722 ± 5.490	0.076
OCT-SRF area (mm^2^)	7.935 ± 5.597	11.786 ± 8.624	4.998 ± 5.545	0.093
OCT-SRF volume (mm^3^)	0.675 ± 0.655	1.119 ± 1.217	0.435 ± 0.650	0.203
CVI	0.36 ± 0.10	0.32 ± 0.11	0.35 ± 0.10	0.800

BCVA, best corrected visual acuity; OCT, optical coherence tomography; OCTA, optical coherence tomography angiography; CMT, central macular thickness; CCT, central choroidal thickness; BNN, branching neovascular network; PED, pigmented epithelial detachment; SRF, subretinal fluid; CVI, choroidal vascularity index. ^$^ Post hoc: *p* = 0.049, between type 2 and type 3. * Post hoc: *p* = 0.440, between type 1 and type 2; *p* < 0.001, between type 1 and type 3; *p* = 0.204, between type 2 and type 3. ** Post hoc: *p* = 0.202, between type 1 and type 2; *p* = 0.022, between type 1 and type 3; *p* = 1.000, between type 2 and type 3.

**Table 2:** Characteristics at 12 months among 3 PCV subgroups.

	Type1 (n = 11)	Type2 (n = 6)	Type3 (n = 15)	*p* Value
BCVA (logMAR)	0.625 ± 0.477	0.561 ± 0.304	0.440 ± 0.337	0.476
OCT-CMT (μm)	226 ± 84	241 ± 52	229 ± 45	0.890
OCT-CCT (μm)	284 ± 96	289 ± 92	289 ± 98	0.993
OCTA-BNN area (mm^2^)	6.057 ± 2.921	3.982 ± 2.362	1.416 ± 1.080	<0.001 *
OCT-PED height (μm)	304 ± 196	351 ± 188	235 ± 162	0.363
OCT-PED volume (mm^3^)	12.378 ± 6.393	16.802 ± 11.961	7.780 ± 8.410	0.111
OCT-PED area (mm^2^)	0.848 ± 0.655	1.616 ± 1.387	0.967 ± 1.850	0.022 **
OCT-SRF area (mm^2^)	0.712 ± 1.400	5.702 ± 5.1	2.786 ± 5.680	0.048 ***
OCT-SRF volume (mm^3^)	0.065 ± 0.099	0.287 ± 0.271	0.193 ± 0.495	0.121
CVI	0.368 ± 0.095	0.383 ± 0.071	0.358 ± 0.072	0.936

BCVA, best corrected visual acuity; OCT, optical coherence tomography; OCTA, optical coherence tomography angiography; CMT, central macular thickness; CCT, central choroidal thickness; BNN, branching neovascular network; PED, pigmented epithelial detachment; SRF, subretinal fluid; CVI, choroidal vascularity index. * Post hoc: *p* = 1.000, between type 1 and type 2; *p* < 0.001, between type 1 and type 3; *p* = 0.053, between type 2 and type 3. ** Post hoc: *p* = 1.000, between type 1 and type 2; *p* = 0.067, between type 1 and type 3; *p* = 0.075, between type 2 and type 3. *** Post hoc: *p* = 0.041, between type 1 and type 2; *p* = 0.774, between type 1 and type 3; *p* = 0.289, between type 2 and type 3.

**Table 3:** Multivariate linear regression analysis for prognostic factors correlated with BCVA at 12 months after anti-VEGF treatment.

	Regression Coefficient	*p* Value
BNN morphology	−0.119	0.466
Baseline BCVA	0.399	0.015 *
Baseline CMT	−0.371	0.023 *
Baseline CCT	−0.127	0.438
Baseline CVI	0.210	0.179
Baseline SRF volume	−0.034	0.832
Baseline SRF area	0.069	0.666
Baseline PED height	−0.108	0.526
Baseline PED volume	−0.050	0.759
Baseline PED area	−0.079	0.626
Baseline BNN area	0.148	0.359
Baseline polyp number	0.224	0.152

BNN, branching neovascular network; BCVA, best corrected visual acuity; CMT, central macular thickness; CCT, central choroidal thickness; CVI, choroidal vascularity index; SRF, subretinal fluid; PED, pigmented epithelial detachment. * Indicated *p* value < 0.05 was considered statistically significant.

## Data Availability

The datasets presented in this study are available from the corresponding author upon request.

## Abbreviations

BNN	branching neovascular network
PCV	polypoidal choroidal vasculopathy
SS-OCTA	swept-source optical coherence tomography angiography
PED	pigment epithelial detachment
SRF	subretinal fluid
AMD	age-related macular degeneration
ICGA	indocyanine green angiography
APOIS	Asia–Pacific Ocular Imaging Society
SD-OCT	spectral-domain optical coherence tomography
FA	fluorescein angiography
BCVA	best corrective visual acuity
VEGF	vascular endothelial growth factor
CMT	central macular thickness
CCT	central choroidal thickness
ETDRS	Early Treatment of Diabetic Retinopathy Study
CVI	choroidal vascularity index
PDT	photodynamic therapy
